# Hedgehog-induced PD-L1 on tumor-associated macrophages is critical for suppression of tumor-infiltrating CD8^+^ T cell function

**DOI:** 10.1172/jci.insight.146707

**Published:** 2021-03-22

**Authors:** Amy J. Petty, Rui Dai, Rosa Lapalombella, Robert A. Baiocchi, Don M. Benson, Zihai Li, Xiaopei Huang, Yiping Yang

**Affiliations:** 1 Department of Medicine and; 2 Department of Pharmacology and Cancer Biology, Duke University School of Medicine, Durham, North Carolina, USA.; 3 Division of Hematology,; 4 Division of Medical Oncology, and; 5 Pelotonia Institute for Immuno-Oncology, Comprehensive Cancer Center, The Ohio State University, Columbus, Ohio, USA.

**Keywords:** Immunology, Cancer, T cells

## Abstract

The programmed death-1 (PD-1) and the PD ligand 1 (PD-L1) interaction represents a key immune checkpoint within the tumor microenvironment (TME), and PD-1 blockade has led to exciting therapeutic advances in clinical oncology. Although IFN-γ–dependent PD-L1 induction on tumor cells was initially thought to mediate the suppression on effector cells, recent studies have shown that PD-L1 is also expressed at high level on tumor-associated macrophages (TAMs) in certain types of tumors. However, the precise role of PD-L1 expression on TAMs in suppressing antitumor immunity within the TME remains to be defined. Using a myeloid-specific *Pdl1*-knockout mouse model, here we showed definitive evidence that PD-L1 expression on TAMs is critical for suppression of intratumor CD8^+^ T cell function. We further demonstrated that tumor-derived Sonic hedgehog (Shh) drives PD-L1 expression in TAMs to suppress tumor-infiltrating CD8^+^ T cell function, leading to tumor progression. Mechanistically, Shh-dependent upregulation of PD-L1 in TAMs is mediated by signal transducer and activator of transcription 3, a cascade that has not been previously reported to our knowledge. Last, single-cell RNA sequencing analysis of human hepatocellular carcinoma revealed that PD-L1 is mainly expressed on M2 TAMs, supporting the clinical relevance of our findings. Collectively, our data revealed an essential role for Shh-dependent PD-L1 upregulation in TAMs in suppressing antitumor immunity within the TME, which could lead to the development of new immunotherapeutic strategies for treating cancer.

## Introduction

The programmed death-1/programmed death ligand 1 (PD-1/PD-L1) axis of interaction is one of the most important immunosuppressive mechanisms within the tumor microenvironment (TME), and targeting this mechanism has led to exciting therapeutic advances in clinical oncology (1–6). While PD-1 was mainly found on the intratumoral lymphocyte population, PD-L1 expression has been observed on a diverse group of cells, including tumor cells, myeloid cells, lymphocytes, and stromal cells (7). Although IFN-γ–dependent PD-L1 induction on tumor cells was initially found and commonly thought to mediate the suppression on effector cells (8), recent reports have shown that PD-L1 is also expressed on stromal cells, especially tumor-associated macrophages (TAMs, refs. 9–11). Additionally, PD-L1 on TAMs is more stable and less dependent on IFN-γ (12). Furthermore, PD-L1 expression was more frequently detected on immune cells than on malignant cells in hepatocellular carcinoma (HCC), non–small cell lung cancer, urothelial carcinoma, and esophageal squamous cell carcinoma (13–15). However, it has been difficult to query the precise role of PD-L1 on each of the cell populations within the TME due to the lack of a conditional ready *Pdl1^fl/fl^* mouse model that allows for lineage-specific deletion of *Pdl1*. Here we showed that we have successfully generated, to the best of our knowledge, the first *Pdl1^fl/fl^* model in pure C57BL/6 background. After breeding to *LysM-cre* mice expressing Cre recombinase in myeloid cells (16), these mice showed a complete deletion of *Pdl1* in myeloid lineage cells. This allowed us to directly study the role of TAM-derived PD-L1 in subverting adaptive immune responses within the TME.

The Hedgehog (Hh) signaling pathway plays an important role in tumorigenesis in many types of human cancer (17). Binding of Sonic hedgehog (Shh), Desert hedgehog (Dhh), or Indian hedgehog (Ihh) to the transmembrane protein Patched-1 on target cells leads to the release of Smoothened (Smo) and activation of downstream signaling events mediated by the Gli family of transcription factors (18). We have recently demonstrated an important role for Hh signaling pathway in promoting M2 polarization of TAMs, leading to a reduction in CD8^+^ T cell recruitment to the TME (19). The immunosuppressive M2 phenotype of TAMs is also closely correlated with PD-L1 expression in several cancer types (20–23). However, what regulates the PD-L1 upregulation on M2 TAMs remains to be determined.

In this study, we first showed that tumor stroma-derived PD-L1 is important for suppression of intratumor CD8^+^ T cells and that the majority of PD-L1–expressing cells in the hepatoma stroma were TAMs. Using a newly generated myeloid-specific *Pdl1*-knockout model, we demonstrated that deletion of *Pdl1* in TAMs rescued intratumor CD8^+^ T cell function and suppressed tumor growth, providing proof for the critical role TAM-derived PD-L1 plays in suppressing intratumor CD8^+^ T cell function. We further found that Hh signaling regulates PD-L1 expression in TAMs and that tumor-derived Shh drives PD-L1 expression in TAMs to suppress tumor-infiltrating CD8^+^ T cell effector function, resulting in accelerated tumor progression. Last, we identified that signal transducer and activator of transcription 3 (Stat3) mediates the downstream effects of Hh in TAMs to regulate PD-L1 expression. Single-cell RNA (scRNA) sequencing analysis of human HCC revealed that PD-L1 is mainly on M2 TAMs, supporting the clinical relevance of our findings. Collectively, our data revealed an essential role for Shh-dependent PD-L1 upregulation in TAMs in suppressing antitumor immunity within the TME, which could lead to the development of new immunotherapeutic strategies for treating cancer.

## Results

### Tumor stroma-derived PD-L1 is critical for suppression of intratumor CD8^+^ T cells.

**Figure 1:**
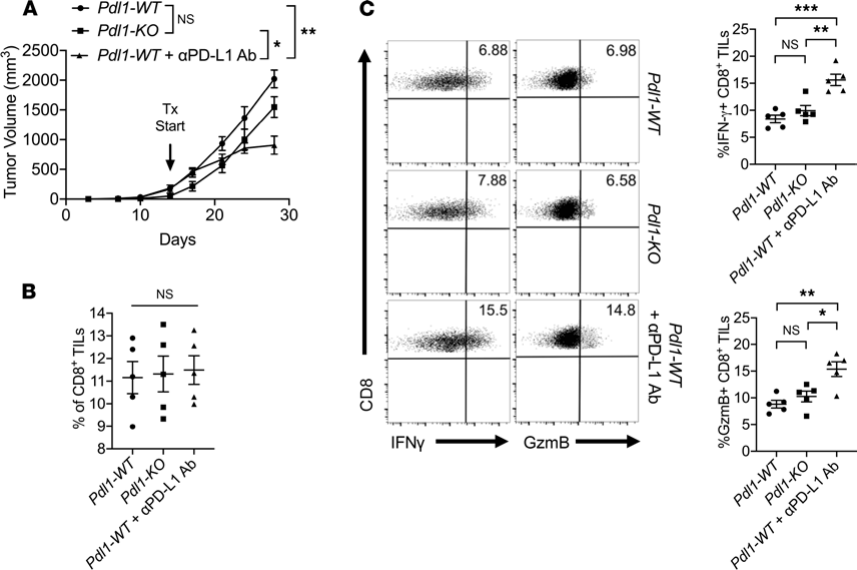
Non–tumor-derived PD-L1 expression is critical for suppression of intratumor CD8^+^ T cells. (**A**) Tumor growth of Hepa1-6 *Pdl1-WT* untreated or treated with 10 mg/kg anti–PD-L1 antibodies and Hepa1-6 *Pdl1-KO* cells subcutaneously inoculated in C57BL/6 mice. Tumor volumes on day 28 at sacrifice are shown. Percentages of intratumor CD8^+^ T cells out of all live cells (**B**) and their productions of IFN-γ and GzmB (**C**) were measured by FACS. Values are mean ± SEM of a minimum of 3 independent experiments. *P > 0.05. **P < 0.005. ***P < 0.0005. *n* = 5 biological replicates per group (**A**–**C**). Kruskal-Wallis test (**A**). One-way ANOVA (**B** and **C**). TILs, tumor-infiltrating leukocytes.

To first investigate whether non–tumor-derived PD-L1 plays a role in suppressing intratumor CD8^+^ T cell function and tumor growth in HCC, we generated mouse hepatoma Hepa1-6 cells with *Pdl1* deletion (referred to as *Pdl1-KO*) using CRISPR/Cas9-mediated gene knockout (24). *Pdl1-WT* Hepa1-6 cells were created using a lentiviral CRISPR/Cas9 vector containing a nontargeting guide RNA (gRNA) sequence. *Pdl1-WT* and *Pdl1-KO* Hepa1-6 cells were subcutaneously inoculated in C57BL/6 mice, and a cohort of mice bearing the *Pdl1-WT* tumor were further treated with 10 mg/kg anti–PD-L1 antibodies 3 times per week starting at day 14 postinoculation. On day 28 at sacrifice, we observed no significant tumor growth reduction (*P* = 0.08) in mice bearing the *Pdl1-KO* tumor compared to *Pdl1-WT* bearing mice. However, treatment of *Pdl1-WT* tumor-bearing mice with anti–PD-L1 antibodies resulted in significant (*P* < 0.005) reduction in tumor growth compared with untreated mice (Figure 1A). Assessment of the tumor-infiltrating CD8^+^ T cells revealed no significant change in CD8^+^ T cell numbers within the tumor stroma regardless of *Pdl1* deletion in tumor cells or with PD-L1–blocking antibodies (Figure 1B). However, CD8^+^ T cells in the tumors treated with anti–PD-L1 antibodies demonstrated a marked increase in effector function measured by IFN-γ and granzyme B (GzmB) production. When compared with the *Pdl1-KO* samples, intratumor CD8^+^ T cells in mice treated with anti–PD-L1 antibodies produced significantly (*P* < 0.05) higher levels of IFN-γ and GzmB measured by fluorescence-activated cell sorting (FACS, Figure 1C). These data suggested that non–tumor-derived PD-L1 plays an important role in subverting intratumor CD8^+^ T cell function.

### TAMs are the main PD-L1–expressing cells within the TME.

**Figure 2:**
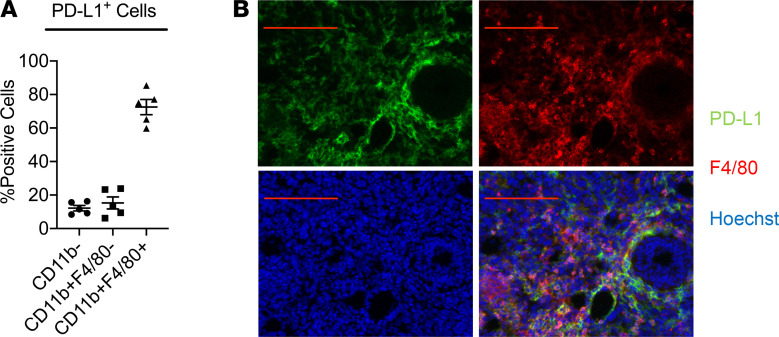
The majority of the PD-L1^+^ cells within the TME are TAMs. (**A**) Percentages of CD11b^–^, CD11b^+^F4/80^–^, and CD11b^+^F4/80^+^ cells within PD-L1^+^ population were assessed with FACS. (**B**) Immunofluorescence staining of PD-L1 (shown in green) and F4/80 (shown in red) in subcutaneously inoculated Hepa1-6 tumors. Scale bar: 100 μm. Values are mean ± SEM of a minimum of 3 independent experiments. *n* = 5 biological replicates per group (**A** and **B**).

We then proceeded to identify the PD-L1^+^ populations within the TME of HCC. We found that 72.3% of PD-L1^+^ cells were CD11b^+^F4/80^+^ TAMs, with CD11b^+^F4/80^–^ other myeloid cells and CD11b^–^ nonmyeloid cells making up 15.3% and 12.4%, respectively (Figure 2A). Immunofluorescence staining of the subcutaneously inoculated Hepa1-6 tumor samples further revealed substantial overlap between PD-L1^+^ cells (shown in green) and F4/80^+^ TAMs (shown in red, Figure 2B). These results indicated that TAMs are the main PD-L1–expressing cells with the TME.

### Generation and characterization of myeloid-specific Pdl1 conditional knockout mice.

**Figure 3:**
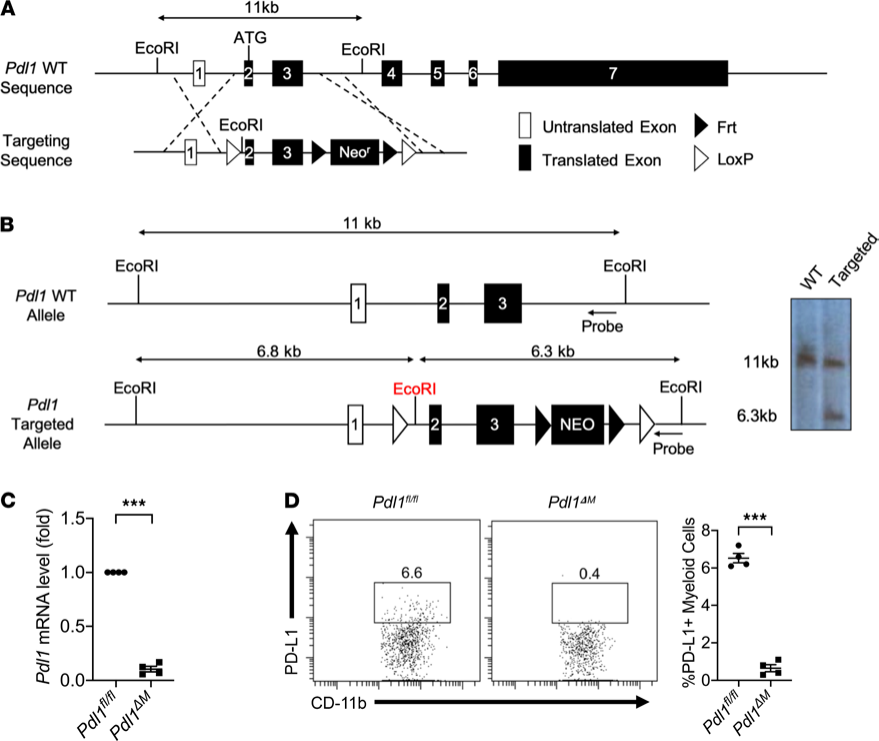
Generation and characterization of *Pdl1^fl/fl^* and *LysM-cre^+^Pdl1^fl/fl^* mice. (**A**) Targeting strategy of generating *Pdl1^fl/fl^* mice. Top line shows WT mouse allele with exons indicated by filled boxes and 5′ untranslated exons indicated by open boxes. Exon 2 contains the translational start site. Second line shows the targeting construct including 11 kb murine genomic arms of homology, a *LoxP* site (hatched triangle) inserted 5′ of exon 2, a Neo^r^ cassette, and a 3′ *LoxP* site. (**B**) Targeted embryonic stem cells were identified by Southern blot analysis of EcoRI-digested genomic DNA using the indicated 3′ external probe. (**C**) Expression of *Pdl1* in *Pdl1^fl/fl^* and *LysM-cre^+^Pdl1^fl/fl^* (*Pdl1*^ΔM^**) BMDMs measured by qRT-PCR. *LysM-cre^+^Pdl1^fl/fl^* was generated by breeding *Pdl1^fl/fl^* with *LysM-cre^+/+^* mice. (**D**) PD-L1 expression on *Pdl1^fl/fl^* and *Pdl1*^ΔM^** peripheral blood cells was quantified by FACS to confirm *Pdl1* deletion in CD11b^+^ myeloid cells. Values are mean ± SEM of a minimum of 2 independent experiments. ***P < 0.0005. *n* = 5 biological replicates per group (**C** and **D**). Two-tailed Student’s *t* test (**C** and **D**).

To study the precise role of TAM-derived PD-L1 in suppressing antitumor immunity in vivo, we generated *Pdl1* conditional ready (referred to as *Pdl1^fl/fl^*) mice in a pure C57BL/6 background. Briefly, *Pdl1^fl/fl^* mice were created by replacing the first 2 coding exons with a new gene segment with exons 2 and 3 plus a neomycin resistance cassette (Neo^r^) flanked by *LoxP* sites via homologous recombination (Figure 3A). Successful integration was confirmed by digesting genomic DNA of embryonic stem cells with EcoRI and performing Southern blotting (Figure 3B). The genotype of the mice was further confirmed by PCR of tail samples (data not shown). We then generated the *Pdl1* conditional knockout mice allowing for myeloid lineage deletion of *Pdl1* by crossing *Pdl1^fl/fl^* mice with *LysM-cre* mice, producing *LysM-cre^+^Pdl1^fl/fl^* (referred to as *Pdl1*^ΔM^**). Deletion of *Pdl1* in myeloid cells in these mice was first assessed by quantitative real-time PCT (qRT-PCR) using bone marrow–derived macrophages (BMDMs) from *Pdl1^fl/fl^* and *Pdl1*^ΔM^** and primers specific for mouse *Pdl1* mRNA (Figure 3C). Knockout status was confirmed with FACS staining of CD11b and PD-L1 on peripheral blood cells from *Pdl1^fl/fl^* and *Pdl1*^ΔM^** mice (Figure 3D).

### TAM-derived PD-L1 is critical for suppressing intratumor CD8^+^ T cell function.

**Figure 4:**
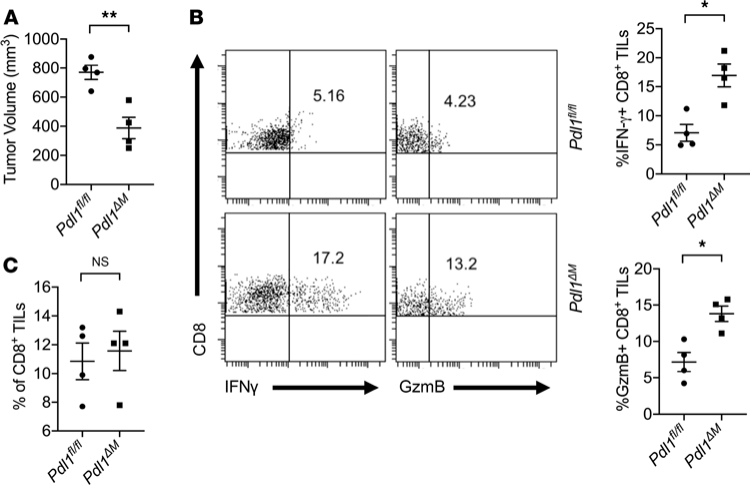
Deletion of *Pdl1* in TAMs rescues intratumor CD8^+^ T cell function. (**A**) Tumor growth of Hepa1-6 cells subcutaneously inoculated in *Pdl1^fl/fl^* and *Pdl1*^ΔM^** mice. Tumor volumes on day 18 are shown. (**B**) Deletion of *Pdl1* in myeloid cells rescued IFN-γ and GzmB productions by intratumor CD8^+^ T cells. (**C**) Percentages of intratumor CD8^+^ T cells quantified by FACS. Values are mean ± SEM of 2 independent experiments. *P < 0.05. **P < 0.005. *n* = 4 biological replicates per group (**A**–**C**). Two-tailed Student’s *t* test (**A**–**C**).

We then proceeded to investigate whether TAM-derived PD-L1 expression affected tumor growth and intratumor CD8^+^ T cell function in vivo. Hepa1-6 tumor cells were inoculated in *Pdl1^fl/fl^* and *Pdl1*^ΔM^**, and tumor growths were monitored over time. We found that *Pdl1*^ΔM^** mice had a significant (*P* < 0.005) reduction in tumor growth, compared with *Pdl1^fl/fl^* tumor-bearing mice (Figure 4A). This was accompanied by a significant (*P* < 0.05) increase in the levels of IFN-γ and GzmB production by intratumor CD8^+^ T cells compared with the control (Figure 4B). However, no significant difference was detected in the percentages of the intratumor CD8^+^ T cells compared to control (Figure 4C). Collectively, these results suggest that PD-L1 expression on TAMs is critical for suppressing intratumor CD8^+^ T cell function.

### Hh signaling regulates PD-L1 expression in TAMs.

**Figure 5:**
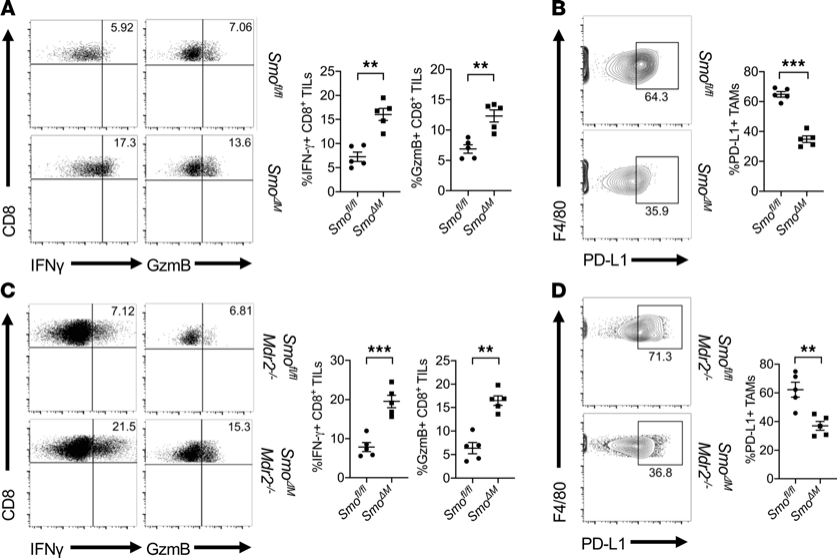
Loss of *Smo* in myeloid cells interferes with PD-L1 expression in TAMs and promotes intratumor CD8^+^ T cell effector functions in vivo. (**A**) *Smo*^ΔM^** intratumor CD8^+^ T cells produced higher levels of IFN-γ and GzmB than *Smo^fl/fl^* TAMs as measured by flow cytometry. (**B**) Active Hh signaling in TAMs resulted in upregulation of PD-L1 expression in subcutaneously inoculated Hepa1-6 tumors. (**C**) Expressions of IFN-γ and GzmB produced by intratumor CD8^+^ T cells were elevated in *Smo*^ΔM^** compared with *Smo^fl/fl^* in an autochthonous *Mdr2^–/–^* HCC model (**F**). (**D**) Active Hh signaling in TAMs resulted in upregulation of PD-L1 expression in the *Mdr2^–/–^* HCC model. Values are mean ± SEM of a minimum of 3 independent experiments. **P < 0.005. ***P < 0.0005. *n* = 5 biological replicates per group (**A**–**D**). Two-tailed Student’s *t* test (**A**–**D**).

Using a conditional knockout mouse model that allows for myeloid lineage deletion of *Smo*, referred to as *LysM-cre^+^Smo^fl/fl^* (*Smo*^ΔM^**), we have shown that deletion of *Smo* suppressed TAM M2 polarization and tumor growth (19). Here we found that intratumor CD8^+^ T cells isolated from *Smo*^ΔM^** tumor-bearing mice produced significantly higher IFN-γ and GzmB (*P* < 0.005) when compared with *Smo^fl/fl^* (Figure 5A), suggesting that Hh-induced M2-polarized TAMs could suppress intratumor CD8^+^ T cell function. Further examination of the expressions of PD-L1, programmed death ligand 2 (PD-L2), CD80, and CD86 on TAMs revealed significantly (*P* < 0.0005) decreased expression of PD-L1 on *Smo*^ΔM^** TAMs compared to *Smo^fl/fl^* TAMs (Figure 5B). No difference was observed with the expression of PD-L2, CD80, and CD86 on *Smo*^ΔM^** versus *Smo^fl/fl^* TAMs (data not shown). To further examine the role of Hh signaling in inducing PD-L1 expression on TAMs in a more physiological environment in which the HCC normally arise, we utilized mice deficient for the multidrug resistance gene 2 (*Mdr2^–/–^*) as an autochthonous model of HCC previously described (25, 26). After generating *LysM-cre^+^Smo^F/F^Mdr2^–/–^* mice (referred to as *Smo*^ΔM^*Mdr2^–/–^*), we also found a consistently increased production of IFN-γ and GzmB in intratumor CD8^+^ T cells isolated from *Smo*^ΔM^*Mdr2^–/–^* tumors (Figure 5C), which was correlated with a reduction of PD-L1 expression on *Smo*^ΔM^*Mdr2^–/–^* TAMs (Figure 5D). Collectively, these results revealed an important role for Hh signaling in promoting PD-L1 expression on TAMs and suppressing intratumor CD8^+^ T cell effector function.

### Tumor-derived Shh ligand is critical for PD-L1 upregulation on TAMs.

**Figure 6:**
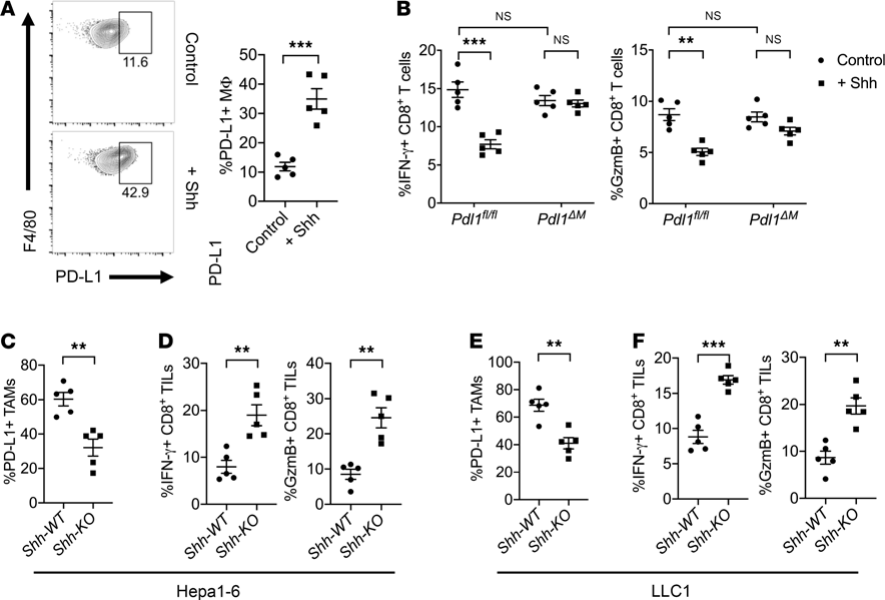
Tumor-derived Shh ligand upregulates PD-L1 expression on TAMs to suppress intratumor CD8^+^ T cell functions in vivo. (**A**) Direct treatment of BMDMs with Shh upregulated PD-L1 expression in vitro. (**B**) Activated CD8^+^ T cells cocultured with *Pdl1^fl/fl^* BMDMs at a 10:1 ratio in the presence of Shh showed suppressed IFN-γ and GzmB production measured by FACS. PD-L1 expressions on TAMs were suppressed in *Shh*-knockout (*Shh-KO*) Hepa1-6 (**C**) and LLC1 (**E**) tumors inoculated in C57BL/6 mice. Expressions of IFN-γ and GzmB produced by intratumor CD8^+^ T cells were decreased in *Shh-WT* Hepa1-6 (**D**) and LLC1 (**F**) tumors when compared with *Shh-KO* tumors inoculated in C57BL/6 mice. Values are mean ± SEM of a minimum of 3 independent experiments. **P < 0.005. ***P < 0.0005. *n* = 5 biological replicates per group (**A**–**F**). Two-tailed Student’s *t* test (**A**, **C**–**F**). Two-way ANOVA (**B**).

To further confirm that Hh signaling can directly promote PD-L1 expression on macrophages, we treated BMDMs from C57BL/6 mice with 5 ng/mL of Shh ligands for 24 hours and measured expression of PD-L1 via flow cytometry. We observed that treatment of BMDMs with Shh significantly upregulated PD-L1 expression (*P* < 0.0005; Figure 6A). Additionally, in vitro coculturing of CD3/CD28-activated CD8^+^ T cells with *Pdl1^fl/fl^* BMDMs in the presence of Shh showed significantly suppressed IFN-γ and GzmB productions (*P* < 0.005) when compared with untreated samples. However, when *Pdl1* was deleted in macrophages, no reduction was observed in the presence or absence of Shh (Figure 6B), indicating the suppressive effects of Shh-induced M2 macrophages on CD8^+^ T cells are mediated through PD-L1.

Furthermore, we have previously generated *Shh-KO* Hepa1-6 hepatoma and LLC1 Lewis lung carcinoma cells using the CRISPR/Cas9 technology to study whether tumor-derived Shh ligands are the major source of Hh signaling within the TME (19). Upon assessing the expression of PD-L1 on TAMs in *Shh-KO* tumor samples, we found that deletion of *Shh* in tumor cells also resulted in reductions of PD-L1 expression on TAMs, both in the Hepa1-6 (Figure 6C) and in the LLC1 (*P* < 0.005; Figure 6E) tumor models. Last, this was correlated with improved intratumor CD8^+^ T cell effector cell functions demonstrated by increased IFN-γ and GzmB productions in the *Shh-KO* samples measured by FACS (Figure 6, D and F). This further supported a critical role for tumor-derived Shh in TAM PD-L1 expression and suppression of intratumor CD8^+^ effector cell functions.

### Hh-induced PD-L1 upregulation in TAMs is mediated by Stat3.

**Figure 7:**
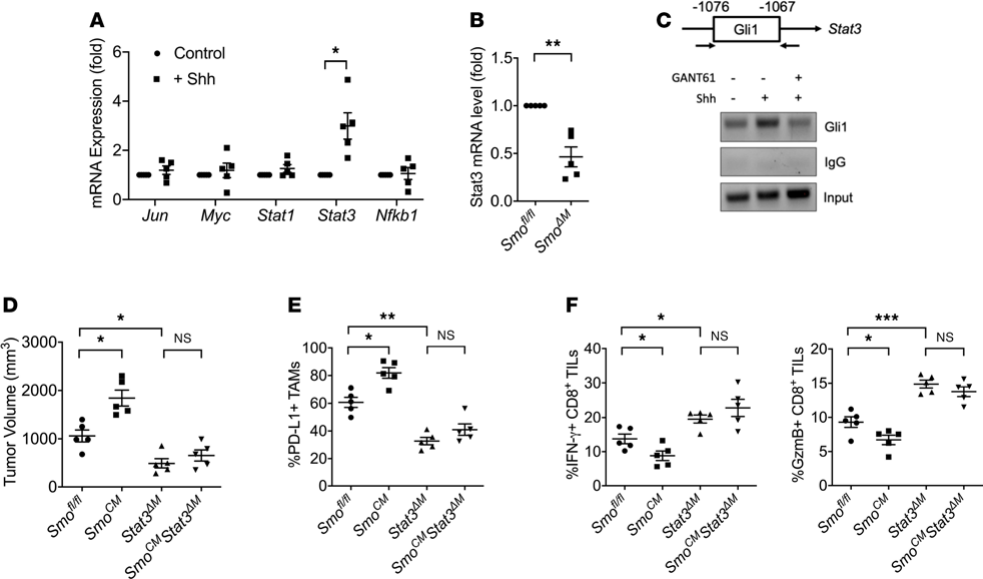
Hh-induced PD-L1 upregulation is mediated by Stat3. (**A**) *Jun*, *Myc*, *Stat1*, *Stat3*, and *Nfkb1* mRNA levels in control and Shh-treated BMDMs were measured by qRT-PCR. Expressions were normalized to β-actin (*Actb*) and compared with control. (**B**) *Stat3* mRNA levels in *Smo^fl/fl^* and *Smo*^ΔM^** TAMs were measured by qRT-PCR. Expressions were normalized to reference gene *Actb* and compared to *Smo^fl/fl^*. (**C**) Gli1 transcription factor was bound to the *Stat3* promoter region in BMDMs treated by Shh as demonstrated by ChIP. Gli1 activity was inhibited using 5 μM GANT61. (**D**) Tumor volumes of Hepa1-6 hepatoma cells subcutaneously inoculated in *Smo^fl/fl^*, *Smo^CM^*, *Stat3*^ΔM^**, and *Smo^CM^Stat3*^ΔM^** mice on day 20 at sacrifice. (**E**) Expressions of PD-L1 on TAMs were upregulated in *Smo^CM^* and decreased in *Stat3*^ΔM^** and *Smo^CM^Stat3*^ΔM^** mice. (**F**) Productions of IFN-γ and GzmB by intratumor CD8^+^ T cells were suppressed in *Smo^CM^* and higher in *Stat3*^ΔM^** and *Smo^CM^Stat3*^ΔM^** mice. Values are mean ± SEM of a minimum of 3 independent experiments. *P < 0.05. **P < 0.005. ***P < 0.0005. *n* = 5 biological replicates per group (**A** and **B**, **D**–**F**). *n* = 3 technical replicates per group (**C**). Two-tailed Student’s *t* test (**A** and **B**). One-way ANOVA (**D**–**F**).

We next sought to understand the mechanisms by which Shh induces PD-L1 expression in TAMs. Previous studies have demonstrated that several transcription factors are associated with PD-L1 upregulation in various cell types, including c-Jun (gene symbol: *Jun*), c-Myc (gene symbol: *Myc*), Stat1, Stat3, and NF-κB (gene symbol: *Nfkb1*; ref. 27). Thus, we surveyed the expression levels of these transcription factors in BMDMs treated with Shh and found that *Stat3* mRNA expression was significantly elevated when compared with untreated control (*P* < 0.05; Figure 7A). We also found that *Stat3* mRNA levels were significantly reduced in *Smo*^ΔM^** TAMs, compared with *Smo^fl/fl^* TAMs (Figure 7B), suggesting that Stat3 could be mediating the downstream effects of Shh in TAMs. Through in silico promoter analysis, we found a consensus Gli-binding sequence (GCCCCGCCCC) at the –1076 to –1067 position upstream of the transcription start site of *Stat3* (28). Using the chromatin immunoprecipitation (ChIP) method, we found increased Gli1 occupancy at that site when BMDMs were treated with Shh. Such binding was reduced to baseline when BMDMs were treated with 5 μM GANT61, a small molecule inhibitor of Gli transcription factors (29), in addition to Shh (Figure 7C). This confirmed that Gli1 transcriptionally regulates Stat3 in macrophages and suggested that Stat3 could be mediating the downstream effects of Shh in TAMs to regulate PD-L1 expression.

To further address this question, we generated 3 additional mouse models: (a) *LysM-cre^+^Smo^C^* (referred to as *Smo^CM^*) mice, which allows for constitutive activation of Hh signaling in *LysM-cre*–expressing cells (30); (b) *LysM-cre^+^Stat3^fl/fl^* (referred to as *Stat3*^ΔM^**) mice that deleted *Stat3* in myeloid cells (31); and (c) *LysM-cre^+^Smo^C^Stat3^fl/fl^* (referred to as *Smo^CM^Stat3*^ΔM^**) that also eliminated *Stat3* in the setting of a constitutively active Hh pathway. After inoculation of Shh-secreting Hepa1-6, we observed accelerated tumor growth in *Smo^CM^* mice compared with *Smo*^ΔM^** (*P* *<* 0.05). *Stat3*^ΔM^** tumor-bearing mice showed significantly reduced tumor growth compared with the *Smo^CM^* mice (*P* *<* 0.05). However, *Smo^CM^Stat3*^ΔM^** mice did not grow larger tumors compared to *Stat3*^ΔM^** (*P* = 0.3; Figure 7D). When comparing PD-L1 expressions on TAMs in these tumor samples to the *Smo^fl/fl^* controls, we found that *Smo^CM^* had the highest PD-L1 expression (*P* < 0.05) and *Stat3*^ΔM^** and *Smo^CM^Stat3*^ΔM^** had the lowest expressions (*P* < 0.005) of PD-L1 with no significant difference between the 2 groups (Figure 7E). Inversely correlated with the PD-L1 expressions, IFN-γ and GzmB levels were observed to be the lowest in the *Smo^CM^* group (*P* < 0.05) and highest in the *Stat3*^ΔM^** and *Smo^CM^Stat3*^ΔM^** groups (*P* < 0.05) when compared with the *Smo^fl/fl^* samples (Figure 7F). Collectively, these results provide evidence supporting that Hh-dependent upregulation of PD-L1 is mediated by Stat3 in TAMs in vivo.

### Human HCC scRNA sequencing revealed expression of PD-L1 in M2 TAMs.

**Figure 8:**
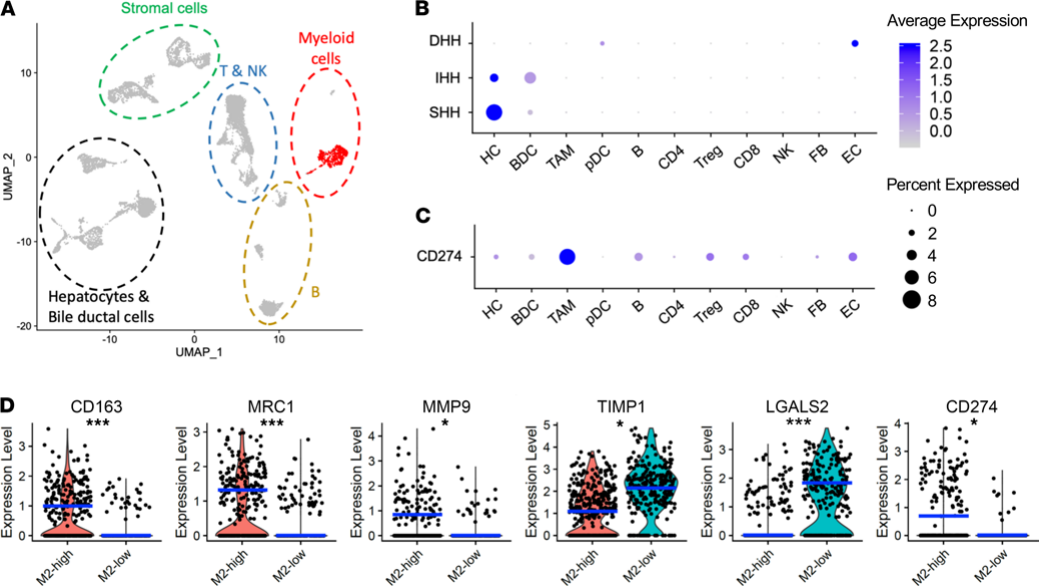
Human HCC scRNA sequencing revealed expression of PD-L1 by M2 TAMs. (**A**) UMAP of 19 human HCC and IHCC scRNA sequencing results. TAMs are highlighted in red within the myeloid cell population. (**B**) Hepatocytes (HC) highly express *SHH* as shown on dot plot. (**C**) TAMs are the dominant contributor to CD274 (PD-L1) expression in human HCC and IHCC. (**D**) PCA of the TAM population revealed correlation between CD274 expression and M2 polarization status of TAMs as shown in violin plots. Blue line represents the median of each population. *P < 0.05. ***P < 0.0005. Two-tailed Student’s *t* test (**D**). LGALS2, galectin-2; MMP9, matrix metallopeptidase 9; TIMP1, tissue inhibitor of metalloproteinases 1.

Last, we analyzed scRNA sequencing results of 19 human HCC and intrahepatic cholangiocarcinoma (IHCC) samples downloaded through the National Center for Biotechnology Information’s Gene Expression Omnibus (GEO) to further query the expression profiles of Hh pathway components and PD-L1 (32). The pipeline of scRNA sequencing analysis has been described previously (33, 34). Briefly, we obtained scRNA transcriptomes of 9493 cells after quality control steps and conducted normalization and principal component analysis (PCA) on the 3000 most variable genes. We then performed uniform manifold approximation and projection (UMAP) dimension reduction analysis, which revealed 24 unique clusters of cells annotated based on known cell lineage–specific markers — including 6 clusters of hepatocytes (HC), 2 clusters of bile ductal cells (BDC), 2 clusters of TAMs, 1 cluster of plasmacytoid dendritic cells (pDC), 3 clusters of B cells, 1 cluster of CD4^+^ cells, 1 cluster of Foxp3^+^ T regulatory (Treg) cells, 2 clusters of CD8^+^ T cells, 1 cluster of natural killer (NK) cells, 2 clusters of fibroblasts (FB), and 3 clusters of endothelial cells (EC; Figure 8A). Analyzing expressions of *SHH*, *IHH*, and *DHH* across all clusters revealed that HCs are the main source of SHH (Figure 8B). More importantly, PD-L1 (gene symbol: *CD274*) was mainly expressed in TAMs (Figure 8C), similar to what we have observed in our mouse models. Further analysis of the TAM population revealed 2 distinctive groups — M2-high and M2-low (Figure 8D). The M2-high group was characterized by higher expressions of *CD163* (scavenger receptor), *MRC1* (CD206, mannose receptor), and MMP9, which are 3 genes associated with the TAM M2 phenotype (35). The M2-low group was defined by higher expressions of *TIMP1* and *LGALS2*, which are associated with antiangiogenic and proinflammatory functions of macrophages in humans (36, 37). *PD-L1* expression was mainly found in the M2-high group, further suggesting that M2-polarized TAMs can contribute to intratumor immunosuppression through PD-L1 in human HCC and IHCC.

## Discussion

In this study, we provided proof that TAM-derived PD-L1 expression is critical for suppressing intratumor CD8^+^ T cell function in vivo. We further demonstrated that Hh signaling regulates PD-L1 expression in TAMs and that tumor-derived Shh drives PD-L1 expression in TAMs to suppress tumor-infiltrating CD8^+^ T cell effector function. Mechanistically, intracellular Hh signaling activated Stat3 to regulate PD-L1 expression in TAMs. Last, analysis of scRNA sequencing results of human HCC samples supported that PD-L1 is mainly expressed on TAMs within the TME and that PD-L1 expression on TAMs is strongly correlated with the M2-TAM phenotype. Thus, our findings identified an important and potentially novel role for paracrine Hh signaling in promoting TAM PD-L1 expression mediated by Stat3, resulting in intratumor CD8^+^ T cell dysfunction and increased immunosuppression within the TME.

Although IFN-γ–dependent PD-L1 upregulation on tumor cells was thought to mediate the suppression on intratumor CD8^+^ T cells in certain tumors (8), recent studies have suggested PD-L1 expression was more frequently detected on immune cells than on malignant cells in HCC, non–small cell lung cancer, urothelial carcinoma, and esophageal squamous cell carcinoma (13–15). However, the precise role of PD-L1 expression on TAMs in suppressing antitumor immunity required further investigation. Here using conditional *Pdl1*-knockout mice, we found that TAM-derived PD-L1 upregulation is critical for suppressing intratumor CD8^+^ T cell function, leading to tumor progression in vivo. In addition, tumor-derived PD-L1 expression was found to be low and noncontributory to intratumor CD8^+^ T cell suppression. This is consistent with a recent report in HCC that tumor environmental factors induce PD-L1 expression on monocytes/macrophages in the peritumor stroma, and high percentages of these PD-L1^+^ monocytes/macrophages are correlated with disease progression and poor survival in patients (9). These previous studies prompted us to create a conditional ready *Pdl1* mouse model that allows us to knock out *Pdl1* in myeloid cells. Using this model, we were able to provide the first definitive proof to our knowledge that PD-L1 expression on TAMs plays a more critical role in suppressing CD8^+^ T cell effector function than tumor-derived PD-L1. This model will also be useful in elucidating the functions of PD-L1 in different cell populations and in various disease processes. It also has important mechanistic and clinical implications as PD-L1 expression pattern may be used to stratify patients for response to PD-1/PD-L1 treatments.

What then regulates the expression of PD-L1 in TAMs? A recent report highlighted that TAMs accumulate in PD-L1^hi^ human HCC tumors and only few of these PD-L1^hi^ samples displayed IFN-γ^hi^ signatures (38), suggesting that there are IFN-γ-independent mechanisms regulating PD-L1 expression in TAMs. Here we revealed that the Hh signaling pathway in TAMs is active and important for upregulation of PD-L1 expression, which has not been reported in previous literature to our knowledge. We also demonstrated that the Shh, produced by tumor cells, is responsible for driving the immunosuppressive phenotype of TAMs characterized by high PD-L1 expression to facilitate its own immune evasion, suggesting the importance of communication between tumor cells and TAMs to promote tumor growth. Together, our study not only revealed the importance of paracrine Hh signaling in modulating the TME to facilitate cancer progression but also identified Shh as a potentially new upstream signaling cascade that regulates PD-L1 expression in TAMs.

How does Shh signaling pathway regulate the expression of PD-L1? Here we provide what we believe is the first evidence to support that Shh pathway transcription factor Gli1 regulates Stat3 expression transcriptionally. Consequently, Stat3 drives the downstream effects of Hh signaling in TAM PD-L1 upregulation, eventually resulting in the functional suppression of CD8^+^ TILs. Stat3 is a key transcription factor that mediates macrophage M2 polarization (39). Murine studies using a myeloid-specific Stat3-knockout model demonstrated the antiinflammatory function of Stat3, characterized by impaired bactericidal activity and increased IL-10 production (40). In tumor studies with HCC, lung carcinoma, and melanoma, it was also shown that inhibition of Stat3 in macrophages abrogated their M2 polarization and protumorigenic effects (41, 42). Furthermore, there is additional evidence indicating Stat3 can regulate the expression of PD-L1 in various cell types (43, 44). Our finding is also consistent with a previous report that PD-L1/2 overexpression was dependent on activation of Stat3 in TAMs in human lymphoma (45). Collectively, our data revealed a potentially novel role for the Shh-Gli1-Stat3 signaling cascade in promoting TAM-derived PD-L1 upregulation and intratumor immunosuppression. However, it remains to be explored whether there is crosstalk between the IFN-γ and Shh pathways through Stat3 or other intracellular mediators in driving PD-L1 expression in TAMs.

Our findings are clinically relevant because we have also shown in our analysis of scRNA sequencing results that human HCC cells produce *SHH* and that *PDL1* (*CD274*) expression is mainly found in TAMs. A query of other human cancers using The Cancer Genome Atlas PanCancer studies revealed that, in addition to HCC, colorectal carcinoma, renal cell carcinoma, pancreatic adenocarcinoma, gastric cancer, and cholangiocarcinoma were the highest *SHH*-expressing cancer types (46). This suggests an exciting therapeutic potential of Hh inhibitors in treating a broad range of human cancers. Indeed, pharmacologic inhibition of Hh signaling with small molecule inhibitors also showed effectiveness in reducing M2 polarization and impressive synergism with immune checkpoint inhibitors in models of HCC and lung cancer in our previous investigation (19). Further investigations are needed to translate this combinational strategy in clinical trials in treating patients with Shh-expressing cancers.

In conclusion, we have identified a critical role for Shh in promoting PD-L1 upregulation on TAMs and that TAM-derived PD-L1 in the TME of HCC is a major and important source for PD-L1/PD-1 axis–mediated suppression on intratumor CD8^+^ T cells. We further demonstrated that Shh-activated TAMs signal through intracellular Stat3, which results in PD-L1 upregulation. Our findings are novel and could potentially provide therapeutic insights in the development of novel chemotherapeutic and/or immunotherapeutic strategies for the treatment of HCC and other Shh-expressing human cancers.

## Methods

### Animals.

Animals used in our studies were described previously (19). In addition, *B6.129S1-Stat3^tm1Xyfu^/J* (*Stat3^fl/fl^*) was purchased from The Jackson Laboratory. Prior to arriving, *Stat3^fl/fl^* mice were backcrossed to C57BL/6 mice in-house for at least 9 generations at The Jackson Laboratory. Backcrossed *Stat3^fl/fl^* mice were crossed with *LysM-cre* mice to generate *LysM-cre^+^Stat3^fl/fl^* mice (referred to as *Stat3*^ΔM^** in this paper) in pure C57BL/6 background. Control mice were *LysM-cre^-^Stat3^fl/fl^* (referred to as *Stat3^fl/fl^*). *LysM-cre^+^Smo^C^Stat3^fl/fl^* (referred to as *Smo^CM^Stat3*^ΔM^**) mice were generated by crossing *LysM-cre^+^Stat3^fl/fl^* with *Smo-M2^C^*.

### Generation of Pdl1^fl/fl^ mice.

A bacterial artificial chromosome clone containing the murine *Pdl1* gene was obtained from a mouse C57BL/6J library (Children’s Hospital Oakland Research Institute), and a targeting vector was designed to flank exons 2 and 3 of the *Pdl1* gene with *LoxP* sites in introns 1 and 3 as follows. A Neo^r^ cassette flanked by 2 *Frt* sites was inserted downstream of exons 2 and 3. A 7.5 kb 5′ homology region, a 1.3 kb 3′ homology region, and a 2.2 kb targeting region containing exons 2 and 3 were subcloned into the PGKneoF2L2DTA vector (a gift from Philippe Soriano, Icahn School of Medicine at Mount Sinai, New York, New York, USA; Addgene plasmid 13445; ref. 47). The construct was confirmed by Sanger sequencing and then introduced into C57BL/6N-PRX-B6N embryonic stem (ES) cells (Primogenix). The neomycin-resistant ES clones were screened for homologous recombinants using PCR primers flanking the 5′ and 3′ recombination sites. Positive clones were subsequently confirmed by Southern blot analysis after restriction digest of genomic DNA with EcoRI and hybridization with a ^32^P-radioisotope–labeled probe against a 772 bp sequence in the intron 3 region of the *Pdl1* gene. ES cells from a confirmed clone were eventually injected into blastocysts derived from C57BL/6 mice, and these blastocysts were transferred to pseudopregnant C57BL/6 females. Chimeric offspring were identified by genotyping using PCR primers flanking the 5′ and 3′ recombination sites. Mice heterozygous for the floxed *Pdl1* allele were mated with C57BL/6 mice for 1 additional generation to ensure germline transmission. Offspring from the additional backcrossing were mated with mice expressing *Cre* under the control of the *LysM* promoter (*LysM-cre*) to generate *LysM-cre^+^Pdl1^fl/fl^* (referred to as *Pdl1*^ΔM^**).

### Cell lines and reagents.

Unless otherwise stated, all cell culture media were obtained from Corning. Hepa1-6 (CRL-1830), LLC1 (CRL-1642), and 293T were obtained from the ATCC. Generation and confirmation of Hepa1-6 *Shh-KO* and LLC1 *Shh-KO* cell lines were described previously (19). Cell lines were cultured in DMEM supplemented with 10% FBS, 100 IU/mL penicillin, and 100 IU/mL streptomycin. TAMs and leukocytes collected from tumor samples were cultured in complete RPMI 1640 medium supplemented with 10% FBS, 1 mM sodium pyruvate, 0.1 mM nonessential amino acids, 50 μM 2-ME, 2 mM l-glutamine, 100 IU/mL penicillin, and 100 IU/mL streptomycin. Recombinant mouse Shh ligand for in vitro treatment of macrophages was purchased from R&D Systems.

### Generation of Pdl1-knockout cell lines using CRISPR/Cas9 technology.

Synthesized guide RNA (gRNA) oligonucleotides (Integrated DNA Technologies) were annealed and subcloned into lentiviral expression vector LentiCRISPR-v2 (a gift from Feng Zhang, MIT McGovern Institute, Cambridge, Massachusetts, USA; Addgene plasmid 52961) for gRNA expression (48). Lentivirus was produced by triple transfection of 293T cells with the gRNA expression LentiCRISPR-v2 vector and the packaging plasmids pCMV-VSV-G (a gift from Robert Weinberg, MIT, Cambridge, Massachusetts, USA; Addgene plasmid 8454) and pCMV-dR8.2 dvpr (a gift from Robert Weinberg; Addgene plasmid 8455) at a 1:1:2 ratio (49). Transfection was performed using Lipofectamine 3000 (Thermo Fisher Scientific) as recommended by the manufacturer. The viral supernatant was collected 48 hours following transfection and filtered through a 0.45 μm filter. Mouse hepatoma Hepa1-6 cells were transduced with lentivirus in the presence of 8 μg/mL Polybrene (MilliporeSigma) for 6 hours. Five days after transduction, transduced cells were single-cell diluted and grown in the presence of 0.8 mg/mL G418 (Thermo Fisher Scientific). Knockout status of expanded single-cell clones was screened with qRT-PCR and confirmed by FACS staining for surface PD-L1. Forward gRNA sequences for CRISPR/Cas9 knockout are as follows: Pdl1 gRNA1: 5′-CACCGGACCGTGGACACTACAATG-3′; Pdl1 gRNA2: 5′-CACCGGATGATCAGCTCCGCTGTG-3′; nontargeting control: 5′-AAGTCTATGCGGGGCTCGTA-3′.

### Tumor models.

Hepa1-6 hepatoma or LLC1 lung carcinoma cells were injected subcutaneously into each mouse in the right hind limb region in 100 μL HBSS with a 27-gauge needle syringe. For treatments, beginning 14 days after injection of tumor cells, mice were injected intraperitoneally with 200 μL (200 μg/mouse) anti–PD-L1 antibody (Bio X Cell) thrice weekly until humane endpoints were reached. Both male and female mice were used. Mice were 6 to 8 weeks of age. There was no systematic means of randomization of mice. Three-digit codes identified the mice, and the experiment was carried out blindly throughout. To estimate the volume of the growing tumor mass, diameters of both the length (*a*) and the width (*b*) of the mass were measured every 3–4 days, after which the tumor volume (*V*) was calculated according to the formula *V* = *ab*^2^/2, as described previously (50). When experimental endpoints were met or when the longer axis of each tumor was more than 20 mm in diameter, all the mice were euthanized according to the NIH guidelines. Tumors were resected and transferred to 2 mL RPMI 1640 medium on ice. Tumor size (mm) was measured with a ruler. The tumors from all experiments were then processed for FACS analysis or sorting on the same day or frozen in O.C.T. Compound (VWR) for cryosectioning.

### Preparation of single-cell suspensions from tumors.

The isolation of TAMs and leukocytes was previously described (19). Briefly, single-cell suspensions were obtained from tumor samples. Leukocytes were further separated from contaminating tumor cells by centrifugation over a 40%–75% Ficoll-Paque (Life Technologies, Thermo Fisher Scientific) gradient at 600*g* for 30 minutes at room temperature. For sorting TAMs, Ficoll-enriched leukocytes were stained with anti-F4/80, anti-CD11b, anti-Ly6G (1A8, BioLegend), and anti-Ly6C (HK1.4, BioLegend) antibodies and purified by FACS for F4/80^+^CD11b^+^Ly6G^–^Ly6C^–^ cells. For sorting CD8^+^ TILs, Ficoll-enriched leukocytes were stained with anti-CD4 and anti-CD8 antibodies and purified by FACS sorting for CD8^+^CD4^–^ cells.

### Isolation of BMDMs.

To prepare macrophages, mice were sacrificed and disinfected with 70% ethanol. Both lower extremities were excised, and the long bones — femur and tibia — were separated from muscular layers and placed in RPMI medium. To extract BMDMs, 10 mL RPMI medium was used to flush out each bone using a 25-gauge needle, and cells were gently dissociated by pipetting. A 70 μm nylon BD Falcon cell strainer was placed atop a 50 mL BD Falcon tube, and the suspension was filtered into the 50 mL tube. The resultant suspension was centrifuged at 300*g* for 5 minutes. The supernatant was then aspirated. ACK lysis buffer (5 mL) was then added, and the contents were incubated for 2 minutes at room temperature. To quench the lysis reaction, 10 mL RPMI was added. The content was then centrifuged at 300*g*, and the cells were washed 2 additional times with 1× HBSS. The cells were plated at 1 × 10^6^ cells per well in a sterile 6-well tissue culture plate in 2 mL BMDM culture medium plus 10 ng/mL M-CSF to obtain mature BMDMs. On day 2, supernatants were aspirated and replenished with fresh culture medium with M-CSF. On day 5, 2 mL fresh culture medium with M-CSF was added. Purity of the BMDMs (>95% F4/80^+^ cells) was confirmed by flow cytometry.

### In vitro coculturing of BMDMs and CD8^+^ T cells.

CD8^+^ T cells were purified from C57BL/6 mouse spleens and cocultured with mature *Pdl1^fl/fl^* and *Pdl1*^ΔM^** BMDMs with or without 5 ng/mL Shh ligands in the presence of 2 μg/mL CD3e and CD28 antibodies (BD Pharmingen clones 145-2C11 and 37.51, respectively) at different ratios and different time points (6, 12, 24, and 48 hours). Data shown here were obtained from the optimal conditions of 10:1 T cell/BMDM ratio and 24-hour coculture period.

### Antibodies and flow cytometry analysis.

Anti-F4/80-PE (BM8), CD4-PE (GK1.5), CD8a-PE-Cy5 (53-6.7), PDL1-APC (10F.9G2), IFNγ-APC (XMG1.2), APC Rat IgG2bκ isotype (RTK4530), and APC Rat IgG1κ isotype (RTK2071) antibodies were purchased from BioLegend. CD11b-PE-Cy5 (M1/70), GzmB-FITC (NGZB), and FITC Rat IgG2aκ isotype (eBR2a) were purchased from eBioscience, Thermo Fisher Scientific. Titration was used to determine the optimal concentration of each antibody prior to each experiment. Cell suspensions were stained with relevant antibodies at 4°C for 15 minutes in PBS with 2% heat-inactivated FBS and 0.1% sodium azide, washed twice, and analyzed with a FACSCanto flow cytometer (BD Biosciences) using FlowJo Software (Tree Star).

### Intracellular cytokine staining.

CD8^+^ T cells were restimulated with 2 μg/mL CD3e and CD28 antibodies for 6 hours in the presence of 5 μg/mL brefeldin A (Invitrogen, Thermo Fisher Scientific). After staining with cell surface markers, the cells were fixed and permeabilized with Cytoperm/Cytofix kit (BD Biosciences) for 20 minutes and incubated with anti-IFNγ-APC and anti-GzmB-FITC antibodies for 45 minutes. The cells were washed twice with perm buffer and analyzed with FACSCanto flow cytometer.

### Quantitative real-time PCR.

Procedures for qRT-PCR were previously described (19). Briefly, total RNA was isolated from sorted cells using TRIzol reagent (Invitrogen, Thermo Fisher Scientific). Reverse transcription was performed with the Superscript First-Strand Synthesis System (Promega). The cDNA samples were diluted 1:10 in water and analyzed in duplicate using SYBR Green Real-Time PCR Master Mixes (Bio-Rad). SYBR green PCR conditions were 1 cycle of 50°C for 2 minutes, 1 cycle of 95°C for 10 minutes, and 40 cycles of 95°C for 15 seconds, 60°C for 60 seconds by a model CFX96 Touch Real-Time PCR Detection System (Bio-Rad). Relative gene expression levels of each respective gene were calculated using the threshold cycle (2^-ΔΔCT^) method and normalized to *Actb* (51). Primer sequences for the following mouse genes are listed: *Pdl1* forward: 5′-GCATTATATTCACAGCCTGC-3′; *Pdl1* reverse: 5′-CCCTTCAAAAGCTGGTCCTT-3′; *Jun* forward: 5′-ACGACCTTCTACGACGATGC-3′; *Jun* reverse: 5′-CCAGGTTCAAGGTCATGCTC-3′; *Myc* forward: 5′-TACCCTCTCAACGACAGCAG-3′; *Myc* reverse: 5′-TCTTGACATTCTCCTCGGTG-3′; *Stat1* forward: 5′-GATCTCTAACGTCTGTCAGCTG-3′; *Stat1* reverse: 5′-GAGGTCCAGGAT TCCTTCGATC-3′; *Stat3* forward: 5′-GGATCGCTGAGGTACAACCC-3′; *Stat3* reverse: 5′-GTCAGGGGTCTCGACTGTCT-3′; *Nfkb1* forward: 5′-GAAATTCCTGATCCAGACAAAAAC-3′; *Nfkb1* reverse: 5′-ATCACTTCAATGGCCTCTGTGTAG-3′; *Actb* forward: 5′-GGTCCACACCCGCCACCAG-3′; *Actb* reverse: 5′-CACATGCCGGAGCCGTTGTC-3′.

### Chromatin immunoprecipitation.

ChIP assays were performed as previously described (19). Briefly, 1 × 10^7^ cells were untreated, treated with 5 ng/mL Shh, or treated with 5 ng/mL Shh + 5 μM GANT61 for 24 hours prior to cross-linking for 10 minutes with 1% formaldehyde. Antibody recognizing Gli1 was purchased from Novus Biologicals. Normal rabbit IgG (Cell Signaling Technology) was used as negative control. Stat3 promoter quantitative PCR was performed with the specific primers flanking the Gli1-binding site (sense: 5′-TGCACGTTTTTCTGCACAAGG-3′; antisense: 5′-AGTTCAAGTTCCAGCATCCCA-3′). PCR products were analyzed with agarose gel electrophoresis.

### Histology and imaging.

Cryostat sections (10 μm) were dried, fixed in cold acetone, and incubated for 30 minutes in blocking buffer (10% goat or rabbit serum in PBS). Slides were then incubated with anti-F4/80–Alexa Fluor 647 (BM8; BioLegend) and anti–PD-L1–Alexa Fluor 488 (MIH5; eBioscience, Thermo Fisher Scientific) in 2% serum in PBS at room temperature in humidity chamber for 30 minutes. After washing 3 times with 2% serum diluted in PBS each for 5 minutes, nuclei were stained with 100 ng/mL Hoechst in PBS and incubated for 15 minutes at room temperature in the dark. Slides were washed 2 times with PBS, mounted with Fluoromount (Southern Biotech), and imaged on a Nikon C2 confocal microscope. Then 405 nm, 488 nm, and 647 nm lasers were used to excite Hoechst-labeled, Alexa Fluor 488–labeled, and Alexa Fluor 647–labeled antigens, respectively. Sequential acquisitions of the multicolor images were used to avoid crossexcitation, and images were overlaid with the Nikon NIS-Element Confocal Microscope Imaging Software.

### Single-cell RNA sequencing data analysis.

The 10x Genomics data set of 19 patient samples of HCC and IHCC were downloaded from GEO (GEO accession GSE125449; ref. 32). Samples were analyzed using Seurat package (version 3.1.1) in R (version 3.6.1) to perform data filtering, normalization, PCA, and UMAP. The standard pipeline of scRNA sequencing analysis was previously reported and slightly modified to fit our analysis (33). Briefly, quality control metrics were used to select cells with mitochondrial gene percentage less than 10% and at least 200 genes detected. The total number of transcripts in each single cell was normalized followed by log transformation. The 3000 most variable genes were further subjected to data scaling and centering. Those variable genes were then used for PCA. The first 30 PCs were applied for UMAP analysis. Data were visualized using ggplot2 (version 3.2.1) in R.

### Statistics.

Results are expressed as mean ± SEM. Comparison between groups was performed by Kruskal-Wallis, Wilcoxon-Mann-Whitney, 1- and 2-way ANOVA, and 2-tailed Student’s *t* test. All statistical analyses were performed with JMP Version 12 software (SAS Software). *P* values of less than 0.05 were considered significant.

### Study approval.

All animal experiments were performed in agreement with the protocols approved by the Institutional Animal Care and Use Committee of Duke University, Durham, North Carolina, USA, and The Ohio State University, Columbus, Ohio, USA.

## Author contributions

AJP designed and performed the experiments, analyzed the data, and wrote the manuscript. RD assisted with performing the experiments and contributed essential ideas and discussion. RL, DMB, RAB, ZL, and XH participated in designing various parts of the study and in discussion and interpretation of the results. YY assisted with designing the experiments, supervised the work, and wrote the manuscript.

## Version 1 03/22/2021

Electronic publication
